# A computational paradigm for real-time MEG neurofeedback for dynamic allocation of spatial attention

**DOI:** 10.1186/s12938-020-00787-y

**Published:** 2020-06-12

**Authors:** Kunjan D. Rana, Sheraz Khan, Matti S. Hämäläinen, Lucia M. Vaina

**Affiliations:** 1 Department of Biomedical Engineering and Graduate Program for Neuroscience, Boston University, Boston, USA; 2 Department of Neurology, MGH, Harvard Medical School, Boston, USA; 3 Department of Radiology, MGH, Harvard Medical School, Boston, USA; 4 Athinoula A. Martinos Center for Biomedical Imaging, Massachusetts General Hospital, Harvard Medical School, Massachusetts Institute of Technology, 149 13th Street, CNY-2275, Boston, MA 02129 USA; 5 McGovern Institute for Brain Research, Massachusetts Institute of Technology, Cambridge, USA; 6 Department of Brain and Cognitive Sciences, Massachusetts Institute of Technology, Cambridge, USA; 7 Present Address: National Institute of Health, Bethesda, USA

**Keywords:** Magnetoencephalography, Neurofeedback, Spatial attention, Decoding, Brain state

## Abstract

**Background:**

Neurofeedback aids volitional control of one’s own brain activity using non-invasive recordings of brain activity. The applications of neurofeedback include improvement of cognitive performance and treatment of various psychiatric and neurological disorders. During real-time magnetoencephalography (rt-MEG), sensor-level or source-localized brain activity is measured and transformed into a visual feedback cue to the subject. Recent real-time fMRI (rt-fMRI) neurofeedback studies have used pattern recognition techniques to decode and train a brain state to link brain activities and cognitive behaviors. Here, we utilize the real-time decoding technique similar to ones employed in rt-fMRI to analyze time-varying rt-MEG signals.

**Results:**

We developed a novel rt-MEG method, state-based neurofeedback (sb-NFB), to decode a time-varying brain state, a state signal, from which timings are extracted for neurofeedback training. The approach is entirely data-driven: it uses sensor-level oscillatory activity to find relevant features that best separate the targeted brain states. In a psychophysical task of spatial attention switching, we trained five young, healthy subjects using the sb-NFB method to decrease the time necessary for switch spatial attention from one visual hemifield to the other (referred to as switch time). Training resulted in a decrease in switch time with training. We saw that the activity targeted by the training involved proportional changes in alpha and beta-band oscillations, in sensors at the occipital and parietal regions. We also found that the state signal that encodes whether subjects attend to the left or right visual field effectively switches consistently with the task.

**Conclusion:**

We demonstrated the use of the sb-NFB method when the subject learns to increase the speed of shifting covert spatial attention from one visual field to the other. The sb-NFB method can target timing features that would otherwise also include extraneous features such as visual detection and motor response in a simple reaction time task.

## Background

The term real-time neurofeedback (rt-NFB) refers to a collection of psychophysiological techniques employed to train individuals to control their own brain activity. This control occurs through self-regulation of behaviorally relevant functional networks and can be implemented to provide self-administered therapeutic interventions [1–6]. Two common non-invasive functional imaging modalities used in rt-NFB training are real-time electroencephalography (rt-EEG) and real-time functional magnetic resonance imaging (rt-fMRI). A recent trend in the rt-fMRI studies is to use multivoxel pattern analysis (MVPA), a neuroimaging technique that decodes brain states (activation profiles that reflect behaviors relevant to a specific brain function) and provides feedback in real-time [7–11]. A disadvantage of rt-fMRI is that it measures the slow hemodynamic response, indirectly related to the millisecond-scale of the neurophysiological activity. Real-time magnetoencephalography (rt-MEG) has also been used in rt-NFB training. MEG has a spatial resolution of about 2 cm on the cortex and has millisecond temporal resolution, making it ideal for situations in which both spatial localization and temporal resolution are desired [12, 13].

In this paper we propose a novel rt-MEG neurofeedback training method, which we refer to as state-based neurofeedback (sb-NFB). This method derives a feedback cue from the recorded changes in brain state based on the output of a fully data-driven pipeline. Unlike previous neurofeedback methods, which target a behavioral feature specific to a specific brain activity pattern, here we use the changes in brain activity (different states) to target temporally defined behaviors, such as the speed of switching spatial attention. The sb-NFB method uses as input oscillatory activity captured by all the MEG sensors. Through using dimensionality reduction techniques, we weighed sensors and oscillatory bands to optimally separate the targeted cognitive states. We used linear support vector machines (SVMs) as the classification tool in MVPA analysis and decoded a cognitive state in real-time from the dataset after we performed dimensionality reduction. The “state” of sb-NFB is the aforementioned cognitive state, representing a behavior, such as attending to a particular spatial location, or preparing to do an action, such as waiting for an auditory cue to press a button. The method we proposed consists of two components. First, the off-line component trains the decoding algorithms. Second, the on-line component applies these algorithms to obtain the time course of a brain state whose time course is then used to extract timing features relevant to the neurofeedback training protocol.

## Results

We present data from five participants who underwent 6 days of rt-NFB training. The sb-NFB protocol was applied in MEG while participants performed the spatial attention switching task. A detailed description of the participants can be found in “Participants” section. The task is presented in “Experimental protocol” and “The feedback cue” sections. The details on data acquisition and preprocessing are described in “MEG data acquisition” section. The mathematical treatment of the problem is presented in “Real-time calculation of time-varying brain state for feedback”, “The training component” and “Sampling from the training period” sections.

The results describe the outcome of the analyses performed for extracting key components of the effects of sb-NFB training on “switch time”, that is the time required to switch spatial attention from one hemifield to another. Specifically, we discuss how the target of NFB training (the switch time) changes during training (“Performance during NFB training” section), the components of the partial least squares (PLS) that are most strongly represented in the neurofeedback (“State-decoding performance” section), and how the spatial pattern changes in order to detect the switch time (“State-decoding performance” section).

### Performance during NFB training

**Fig. 1:**
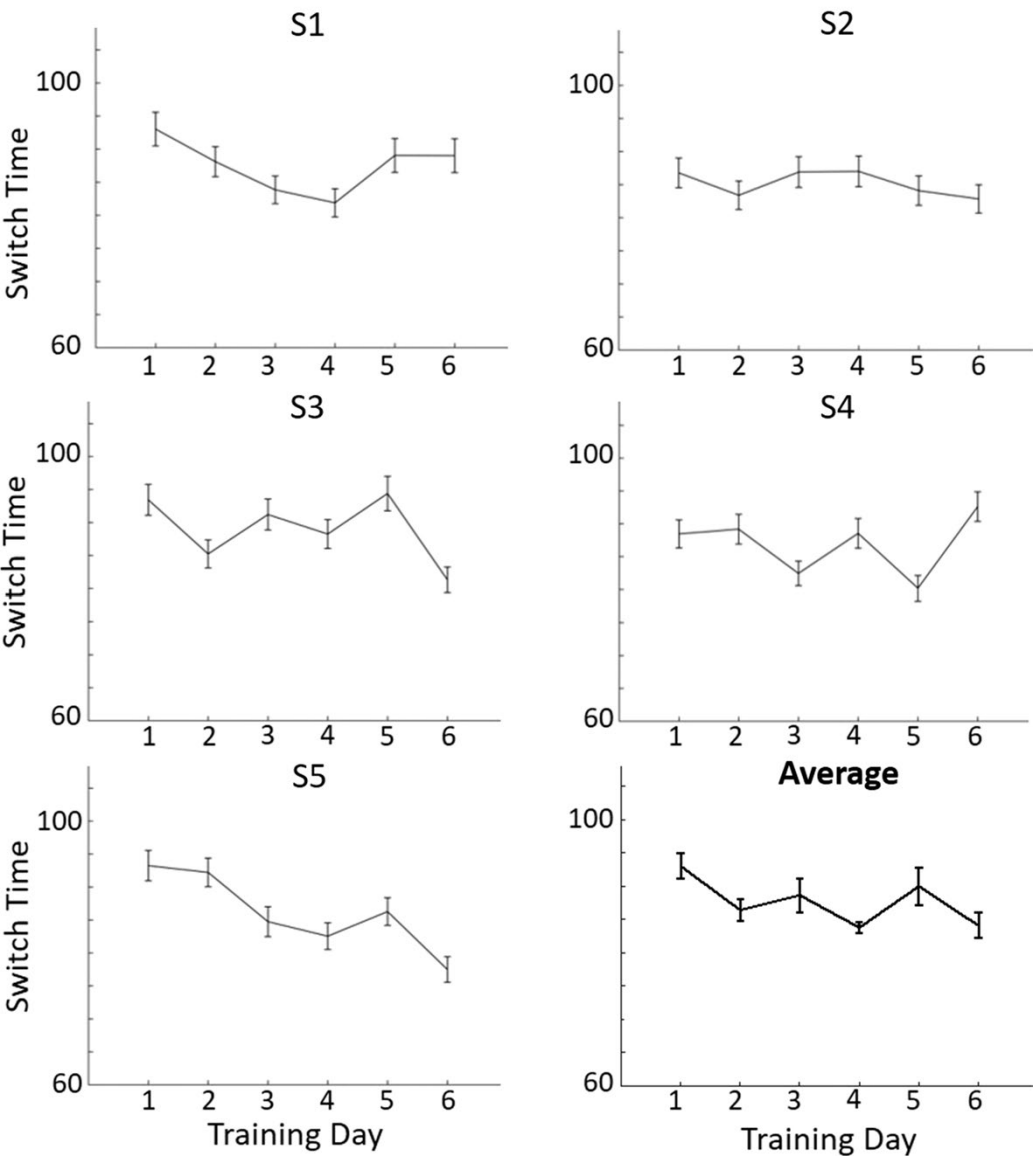
Switch time across the 6 days of sb-NFB training for each of NFB subjects S1 to S5 and the average over subjects. For each subject, average switch time is plotted for each day of training. The error bars represent 1 standard error above and below the mean across trials. The bottom right figure represents the average switch time across the 5 subjects on each day with error bars representing 1 standard error across subjects

In Fig. 1, we show the performance of the five subjects who participated in neurofeedback training. Note that even subject S4 who did not consistently improve did have a trend to reduce the switch time except on the last day.

### State-decoding performance

This section illustrates the sensor weighting components used in the switch time calculation for the NFB cue. Here, the PLS regression computation is used to compute weights to determine attention location. We show the patterns of sensor-level data that resulted in classifying the left and right attention state. The sb-NFB algorithm produced a set of orthogonal “components” consisting of weights on all 306 sensors in each of the frequency bands as described in “The training component” and “Sampling from the training period” sections (alpha, beta, and gamma). The gamma-band, due to its high frequency and thus volatility compared to lower frequencies of the alpha and beta bands, did not result in an informative classification information (less than 5% discrimination power in the first component).

**Fig. 2:**
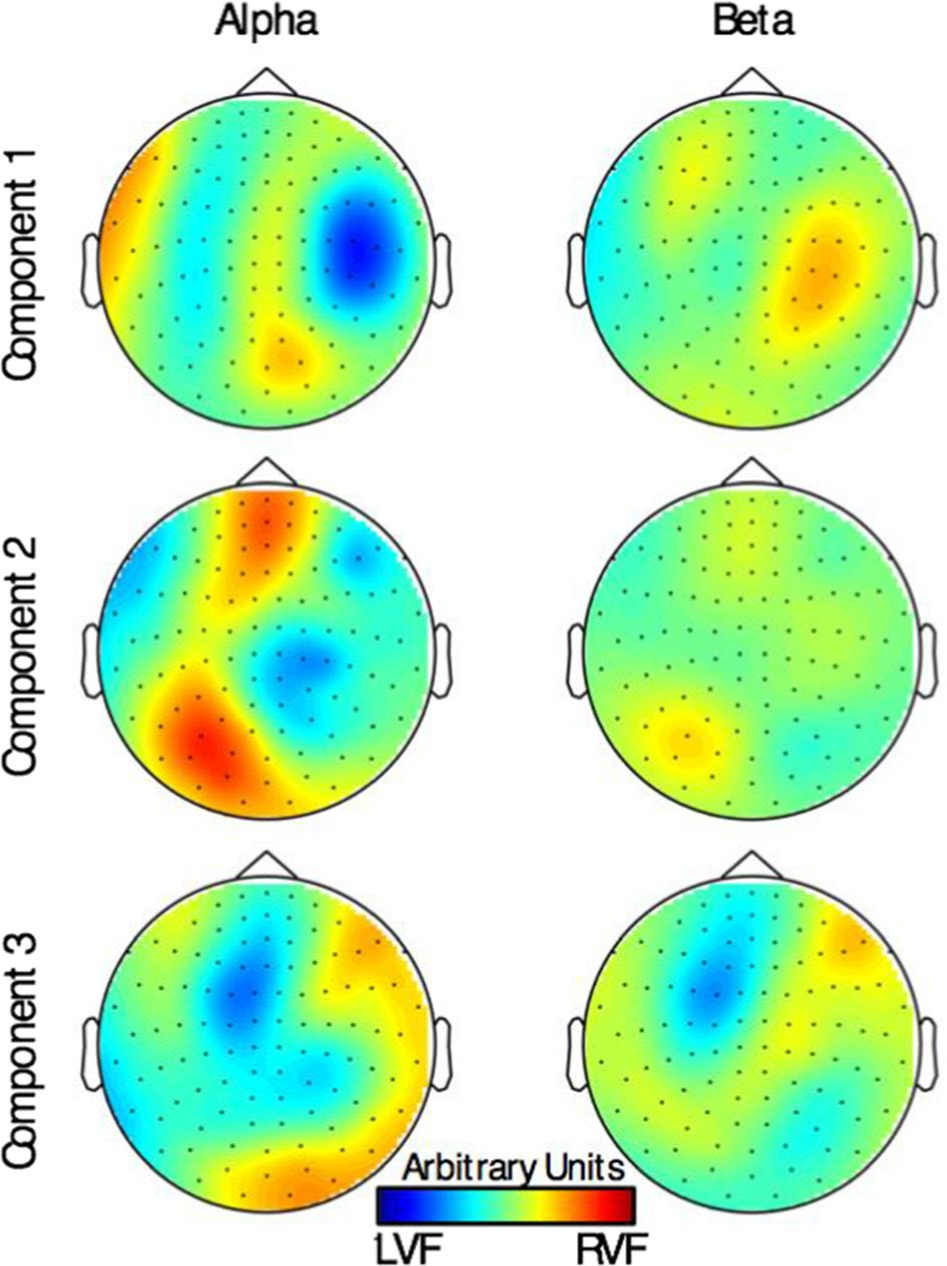
Magnetometer sensor weighting across the sensor cap for the first three components resulting from the PLS regression. The color heatmaps show the weighting of magnetometer sensor measurements along the first three PLS dimensions used to classify visual attention directed to the left or right visual fields. Areas in blue indicate sensors (represented as black dots over those areas) that are higher in power when attending to the left visual field (LVF), whereas areas in red comprise sensors that are higher in power when attending to the right visual field (RVF)

The sensor weights were either positive or negative. The sign of sensor weights suggests that the algorithm identified a pattern of activation where high power in the positively weighted sensors was correlated with low power in the negatively weighted sensors. We monitored the changes to these patterns over the course of training, to determine whether the pattern was maintained or strengthened with training. Figure 2 shows the maps of the weights of the magnetometer channels in our MEG (see “MEG data acquisition” section) after PLS, prior to the SVM separation. The first three components, which comprised 76% of the weights in the alpha and beta bands are illustrated.

In the first component, the alpha-band has strong power in the right hemisphere occipital sensors (yellow) anti-correlated with the right the hemisphere parietal and temporal sensors (blue). In the right hemisphere, when attention is allocated to the left visual hemifield, we see anti-correlation in the parietal and temporal sensors between the alpha and beta bands. Thus, we conjecture that the beta power is high when the alpha power is low, and that the strength of the alpha/beta ratio discriminates between attention to the left or the right visual hemifield. However, the Pearson correlation of the projection of this component onto the sensor data across training days did not reveal significant change over the course of training (*ρ* = − 0.203, *p* = 0.282).

In the second component, there was significant involvement of the occipital and parietal sensors in the alpha-band. The alpha power in the left and right hemisphere sensors was anti-correlated. There was a significant and correlated component of the alpha power in the frontal sensors straddling both hemispheres, suggesting that the frontoparietal and occipital-parietal sensors activation are related. The Pearson correlation showed a weak trend of decrease in the significance of the second component over the days of training (*ρ* = − 0.341, *p* = 0.065), suggesting that the occipital activation correlated with the frontal activation, decreased with training.

In the third component, sensors activations in both the alpha and beta frequency bands were mostly prefrontal in both the left and the right hemispheres. However, some contribution of alpha power was also seen in occipital sensors. Pearson correlation did not show a significant correlation between the activations in the third component and the day of training (*ρ* = 0.173, *p* = 0.360). Taken together, these results suggest that the activation patterns between different cortical areas were necessary to best separate LVF and RVF.

### State-decoding performance

**Fig. 3:**
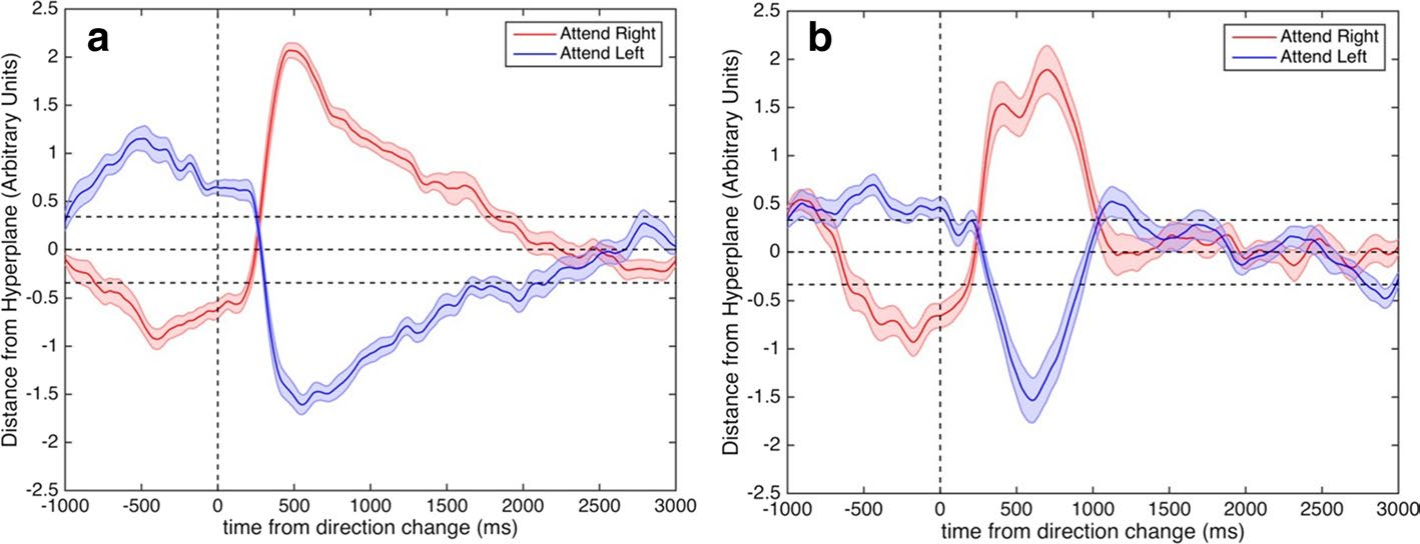
Decoded state signal. **a** Averaged state signal for trials with switch when initially attending to the right (red) and initially attending to the left (blue) visual field in S1. **b** Averaged state signal for trials with switch when initially attending to the right (red) and initially attending to the left (blue) visual field in S2. The x-axis denotes the time relative to the change in motion direction. Time zero denotes the time when the attention switch occurred. The y-axis denotes the distance from the separating hyperplane from the support vector machine in arbitrary units. The goal of the distance measure is to represent separability of where spatial attention is directed in one dimension (along the perpendicular to the separating hyperplane). Positive values indicate that covert attention is directed to the LVF and negative values indicate the covert attention is directed to the RVF. Horizontal dotted lines indicate 2 standard deviations above and below zero

The sb-NFB method must detect the shift of attention within a single trial. In this task (described in detail in Appendix), the subject covertly switched attention from the left to the right or from the right to the left visual field in 80% of trials. The mean and standard error of state signals related to attention shifts are shown in Fig. 3a, b for participants S1 and S2, respectively, time-locked to the change in motion direction in the attended aperture. The other 20% of trials had no switch of attention and therefore were not included in the data analysis.

Figure 3a, b reveals a noticeable delay from the cue to the action of completing the switch of attention between one visual hemifield to the other. We quantified the delay by finding the time between cue presentation and the time of crossover of the threshold line. For S1, the delay was 223 ms ± 79 ms, and for S2, the delay was 209 ms ± 133 ms.

## Discussion

In this study, we propose a state-based neurofeedback (sb-NFB) method for rt-MEG neurofeedback training. The sb-NFB method decodes the timings related to brain state within single trials using oscillatory activity separated into frequency bands. The goal of this method is to train cognitive behaviors which cannot be directly measured through purely behavioral techniques. For example, here we measured the time to switch attention from one spatial location to another. If instead we measured the reaction time to press the button, that measure would indeed include the targeted switch time, but it would include as confounding factors the time involved in detecting the cue as well as the time to button press. Therefore, behavioral feedback training on reaction time may lead to improvement on how fast the subject detects the cue or how fast the subject presses the button instead of improving the speed of the spatial attention switch. The present method is entirely data-driven, that is it makes no assumptions on activation patterns related to the targeted brain states. The unique activation profile of each subject may be used as a basis for developing personalized neurofeedback training protocols.

This method is different from EEG-based neurofeedback, but employs frequency-based techniques from this domain. EEG-based neurofeedback studies have demonstrated that higher activity within specific frequency bands is associated with performance on a specific cognitive task. For example, Vernon et al. [14] sought to evaluate NFB-derived performance on semantic working memory or attention. In three subject groups, performance on semantic memory and attention was examined prior to initiating NFB training. During NFB training, the first group was instructed to enhance theta (4–7 Hz) activity and simultaneously inhibit activity in delta (0–4 Hz) and alpha (8–12 Hz) bands. They found that NFB positively influenced performance on working memory, which is associated with the theta band [15–17]. The second NFB training group was instructed to enhance sensorimotor rhythm (SMR) activity (12–15 Hz), while simultaneously inhibiting theta (4–7 Hz) and beta (18–22 Hz) bands. These results suggest that rt-NFB training positively influenced performance on sustained attention and memory.

A drawback of NFB based on standard clinical EEG (10–20 system) is that activation can only be localized to cortical regions at a lower spatial resolution [18, 19]. MEG has higher localization accuracy than typical EEG configurations, though localization accuracy is comparable between high density EEG and MEG [20]. Real-time functional magnetic resonance imaging (rt-fMRI) on the other hand can measure target activations localized to a millimeter scale anywhere across the brain volume [1, 4, 6, 21]. The disadvantage of rt-fMRI compared to rt-MEG/EEG is that it cannot capture the dynamic activity discussed here, due to feedback at the time scale of seconds. Another drawback of EEG is that with a large number of electrodes there is a long preparation time in EEG compared to MEG [22].

A number of EEG- or MEG-based neurofeedback studies have targeted at attention or other cognitive functions. However, these studies did not include “dynamic” features, such as the speed at which the subject switches spatial attention, which is the goal of sb-NFB. Instead, these studies focused on enhancing “static” features, such as alertness or lateralization of spatial attention. For example, Egner et al. [23, 24] used EEG to train the increase of the ratio of the sensorimotor rhythm (SMR) power to the beta rhythm power in healthy participants for the purpose of augmenting alertness. They differentiated between errors of omission, or mistakes due to perceptual error or misunderstanding, and errors of commission, or mistakes due to incorrect button presses and impulsiveness. The results showed that participants who trained successfully to increase this ratio had fewer errors of commission [23, 24]. Barnea and colleagues [25] trained the ratio of SMR power to theta rhythm in EEG on the performance of an attention network test [26]. They found that targeting different sensors led to changes in behavior related to alertness, spatial orienting, and executive control [25]. In MEG one study trained alpha-band lateralization across occipital sensors and showed that it enhanced the subjects’ ability to modulate their alpha-band lateralization at will and that such modulation affected the ability to discriminate faces presented in the left or right visual field [12]. Using the Posner cueing task [27], another investigation trained the alpha modulation index (AMI, the normalized alpha-band power difference between attention to the left and right visual fields) over occipital sensors [13]. They reported that NFB training on the AMI affected attentional bias in the Posner Cueing task.

Using the SAST task we applied the sb-NFB method to train the time required for the spatial shift of covert spatial attention. The ability to quickly switch spatial attention is necessary for many everyday tasks. For example, in a volleyball game, players must switch attention between players and the ball. While driving a car, one needs to attend to and switch attention between the other cars on the road, stop-lights, bicycles, and pedestrians. At the beach, lifeguards must monitor numerous swimmers and switch spatial attention between them to respond immediately to signs of drowning.

The study demonstrates an sb-NFB pipeline which targets temporally defined features. The neurofeedback training presented here showed decrease in switch time across subjects. It is possible that with further training, saturation level would be reached, which would be more effective in characterizing the achievable improvement in sb-NFB training. A control for sb-NFB training was not implemented to compare against the decrease in switch time. After this proof-of-concept demonstration of sb-NFB we plan to run a new study with a new set of subjects, perhaps with a sham-NFB cue in MEG, or with a related behavioral cue, such as reaction-time. With the data presented here we were able to show that we could effectively measure and monitor the decrease in switch time over a number of training days in multiple subjects. This demonstrates the efficacy of this pipeline for monitoring and feeding back the switch time.

## Conclusion

In this paper, we showed that the state-based neurofeedback method (sb-NFB) could be used to decode and train on timing-related features. For the sb-NFB method to be an effective tool for studies of cognitive enhancements and to be useful in clinical applications, we need to demonstrate that the learned self-regulation is maintained after the end of the training period and that it transfers to situations where neurofeedback is not available. Furthermore, we will also investigate how subjects’ performance and changes in the patterns of cortical functional connectivity before and after training compare between training with the sb-NFB methods versus behavioral training (perceptual learning).

## Methods

### Participants

Five individuals (4 female; M = 24.3 years, SD = 5.1 years) participated in this study. All participants were naïve to the purpose of the study and had not previously participated in a perceptual learning or an rt-NFB study. All had previous experience with psychophysical tasks. An essential inclusion criterion was that subjects were able to maintain stable fixation throughout an entire trial during the experimental task. This was evaluated by the experimenter during a preliminary session where subjects were administered 20 example trials before the start of the experiment. All participants were right-handed, had no known neurological or psychiatric disorders, had normal or corrected-to-normal vision, and were not red-green color blind. They gave written informed consent in accordance with the Declaration of Helsinki and the procedures and protocols approved by the local Ethics Committees on Human Research at Boston University and Massachusetts Institute of Technology (MIT). Task examples were administered at Boston University. The neurofeedback training in MEG was conducted at the MIT McGovern Institute for Brain Research.

### Experimental protocol

**Fig. 4:**
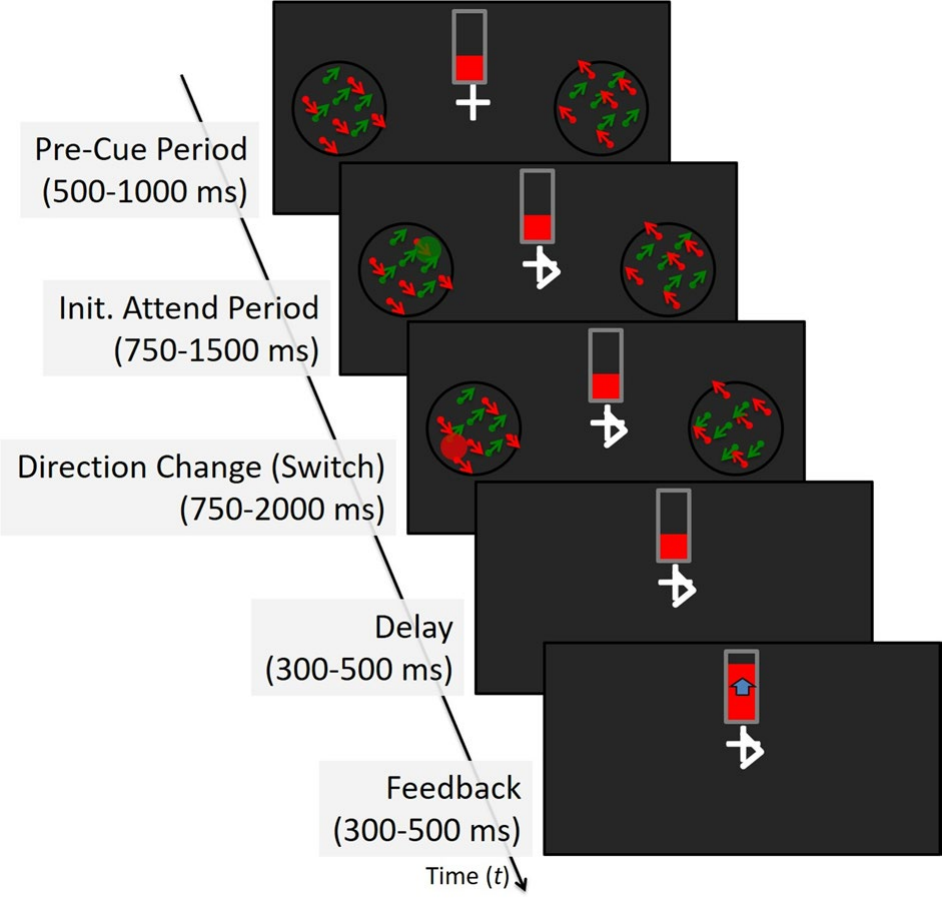
The NFB task. At the beginning of each trial, a fixation mark is shown at the center of the screen. Participants are asked to maintain fixation at the center of the screen (fixation cross/arrow) for the full duration of each trial. A feedback thermometer was displayed just above the fixation mark, with height (indicated in red) representing the speed of attention switch in the previous trial. On the left and right side of the screen were displayed two apertures equidistant from the fixation mark. Each aperture contained a random dot kinematogram (RDK) consisting of an equal percentage of red and green dots. In each aperture, the dots moved in diagonal planar motion with the green dots moving orthogonally to the red dots. During the “Init. Attend Period”, an arrow was superimposed on the fixation cross, which indicates the hemifield that the participant was required to attend. The arrow sign was obtained by adding two skewed lines on one horizontal end of the fixation cross, thus keeping the center of the fixation mark unchanged. The subjects continue to fixate the intersection point of the two perpendicular lines of the cross when the arrow was present and not move the eyes to the attended hemifield. At the same time, a disc superimposed on the RDK was displayed in the opposite aperture and was set to change location and color (red or green) at random time intervals throughout each trial. After a variable period of time, the RDK (red or green) in the attended aperture may change direction; if the attended dots change direction, the participant must switch attention to the aperture in the opposite hemifield and as quickly and as accurately as possible indicate by a button press the color of the disk (red or green). The two apertures are removed from the screen concurrently with the participant response (or if no response is entered after a delay of 300–500 ms) and the height of the thermometer is updated to reflect the switch time of the previously completed trial. In the case of catch trials (when the direction of motion in the attended RDK does not change) the height of the thermometer does not change

The purpose of the NFB training was to decrease the time that subjects required for switching spatial attention from one visual field to the other over multiple consecutive days of training in MEG. The stimulus shown schematically in Fig. 4 is described in detail in Appendix. Before the beginning of the experiment, participants were instructed to pay attention to either red dots or green dots. They were also asked that for the duration of each trial they maintained their fixation on the cross-hair white fixation mark presented at the center of the display. The stimulus began by simultaneously displaying in the right and left visual field, in two circular apertures two random-dot kinematograms (RDK) consisting of red and green dots. The center of each aperture was presented at 8 degrees eccentricity from the central fixation mark. The red and green dot patterns were superimposed on each other and moved diagonally and orthogonally to each other. The superimposed patterns made attending to the aperture necessary to perceive a change in direction to the specified dot pattern. After 500–1000 ms from the beginning of the trial, a white arrow, pointing to the left or right, was superimposed onto the fixation mark, indicating which RDK (in the left or right visual field) to attend to. As soon as subjects perceived the direction change of the attended dots, they had to switch attention to the aperture displayed in the non-attended (opposite) visual field (during the “switch” window). As soon as the arrow was displayed, a small disk of either a faint red or green color was superimposed on the RDK in the location to be switched to. Throughout the attend and switch windows the disc briefly changed color (e.g., from red to green) at random times and was displayed at random locations within that aperture. The participants’ task was to respond as soon as possible, via a button box press with the right hand, to the color of the disk after they have shifted attention to that visual field. Of the total 80 trials in a block, in 16 (catch trials) the direction of the attended dots motion did not change, and thus in the absence of the cue to switch attention, the participants were instructed to continue attending to the motion until the end of the trial when the screen went blank.

### The feedback cue

After either the participant responded or the trial ended, the feedback was presented as a vertical, red thermometer situated 2° above the fixation mark. The thermometer had a maximum height of 3°, a width of 1°, and luminance of 40 cd/m^2^. It was surrounded by a grey border of 0.1° thickness and luminance of 35 cd/m^2^. The height of the thermometer was based on $z_{t} = {{\left( {\rho - \mu_{\rho } } \right)} \mathord{\left/ {\vphantom {{\left( {\rho - \mu_{\rho } } \right)} {\sigma_{\rho } }}} \right. \kern-0pt} {\sigma_{\rho } }}$, the $z$-scored switch time, where $\mu_{\rho }$ is the mean switch time and $\sigma_{\rho }$ is the standard deviation of switch times from the previous block of trials. The computation of $\rho$ will be explained in “The training component” and “Sampling from the training period” sections. A 0.5° deflection in the thermometer was set to correspond to $\Delta z_{t} = 1$ with $z_{t} = 0$ at the middle so that the range of the thermometer, whose height was 3° was $z_{t} = - 3 \ldots 3$.

### MEG data acquisition

The magnetoencephalography (MEG) study was conducted at the Athinoula A. Martinos Imaging Center at MIT’s McGovern Institute for Brain Research. Participants were seated in a chair under the MEG sensor array and faced the projection screen placed at a distance of 138 cm. The task stimuli, see “Experimental protocol” and “The feedback cue” sections, were projected onto a 44” back-projection screen through an aperture in the MEG chamber using a Panasonic DLP projector (Model #PT-D7500U). During the experiment, the room lighting was dimmed.

The MEG data were acquired with a 306-channel Neuromag Triux whole-head MEG system (Elekta-Neuromag, Finland), comprising 102 pairs of planar gradiometers and 102 magnetometers. The system was housed in a three-layer magnetically shielded and sound-proof room (Ak3b, Vacuumschmelze GmbH, Hanau, Germany). In the rt-NFB task discussed here, the MEG sensor data were segmented into trials, − 1000 … 3000 ms relative to the onset of motion direction change in the attended aperture. The data from the catch trials, where the direction of motion did not change, were discarded from the analysis.

The data were transmitted between the acquisition workstation to the stimulus workstation using Fieldtrip real-time buffer [28]. Data were transmitted in 100 ms pieces and were assembled on the stimulus workstation. Subsequently, to attenuate environmental noise a signal-space projection (SSP) operator constructed using the singular value decomposition (SVD) of the empty room MEG data was applied to the data at the stimulus machine [29].

### Real-time calculation of time-varying brain state for feedback

The aim of the sb-NFB method we propose is to train the temporal dynamics of the brain state, corresponding to the timing when a targeted cognitive behavior begins, ends, or changes. In this section, we describe the process of defining and detecting the brain states reflecting cognitive behaviors targeted by the training.

**Fig. 5:**
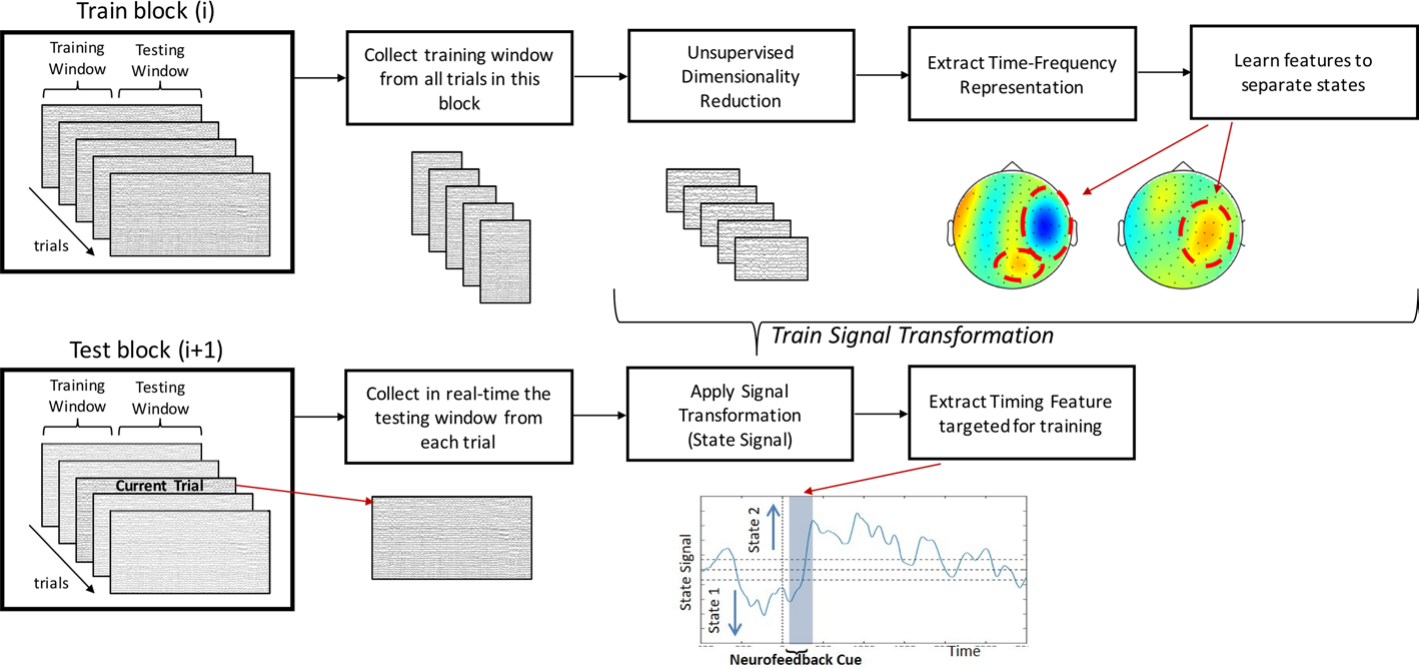
State-based neurofeedback (sb-NFB). During training (left), the training window from a block of trials (block i) is used to train the signal transformation, which is tested during the development window. The training component involved unsupervised dimensionality reduction using the data from the training window, extracting the time–frequency representation of these periods, and then learning features that best separate the states. During state decoding (right), each trial from the current block (block i + 1) is collected in real-time and transformed using the trained signal transformation from the previous block to obtain a state signal (bottom right plot). During testing, the features from the training are used to detect attention state over time, from which we extract the timing feature of interest. The timing feature targeted for training was extracted from the state signal for use as the NFB cue

To decode a brain state, the decoding algorithm is trained on a set of MEG sensor data. Therefore, sb-NFB requires a time period in which the brain state is known (referred to as the training window in Fig. 5). Following conventions of the machine-learning literature, we refer to the time period in which the brain state change is measured, such as a shift of attention from one visual field to the other, as the development window. Due to the computational complexity (1530 dimensions) involved in training the decoder, the training is conducted between the testing blocks. This is the training component of the sb-NFB method. It results in a signal transformation that best separates the features of interest. The outcome of the signal transformation is used in the state decoding component to transform raw MEG signal data into a state signal that represents the brain states targeted by the sb-NFB.

### The training component

Using data from the training period, the signal transformation was trained as follows: (i) the dimensionality of the data was reduced by an unsupervised dimensionality reduction step, and (ii) the features that optimally separate the reduced dimensionality dataset were extracted.

### Sampling from the training period

In the algorithm, a fixed set of samples was selected from the training period and were labeled with the corresponding brain state. In our implementation, we randomly selected 3 samples during the training period for each trial. We used a 200 ms buffer after the cue was presented to ensure that the subject is provided sufficient time after seeing the cue to attend to the target side of the visual field to obtain samples.

### Dimensionality reduction

The dimensionality reduction is necessary due to the high dimensionality of the dataset (306 sensors). The amount of training data required for the feature extraction is proportional to the dimensionality of the data. Reducing dimensionality implies that fewer trials are needed to train the signal transformation. We applied principal component analysis (PCA) to the raw MEG sensor measurements. PCA transforms multivariate data into a set of orthogonalized components (“PCA space”) ordered by their contribution to the signal power.

**Fig. 6:**
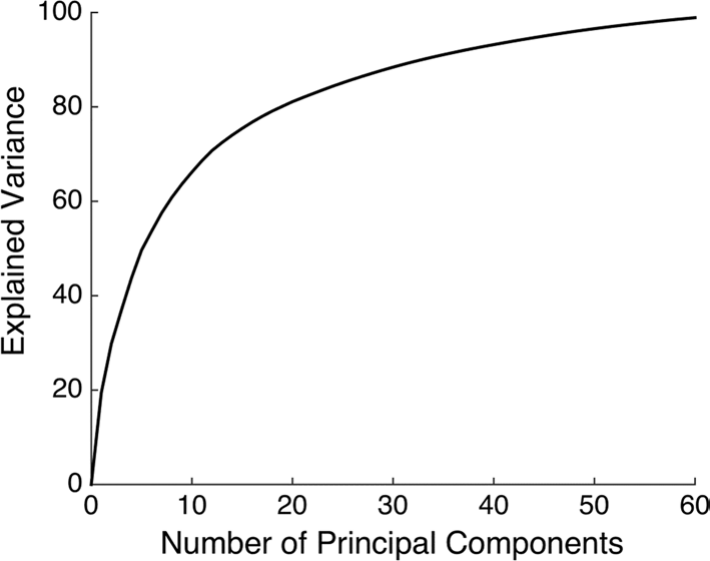
Amount of variance of data explained by a given number of PCA components

To reduce the dimensionality of the $m = 306$ dimensional MEG data with $T$ time samples

$$ X = \left[ {x_{1} , \ldots ,x_{T} } \right] \in {\mathbb{R}}^{m \times T} $$,

we arrange the first orthogonal $k$ PCA components as columns of a matrix $P_{k} \in {\mathbb{R}}^{k \times m}$. We then form the $k \times T$ dimensional

$$ Y = P_{k}^{T} X $$

,where due the properties of the PCA the matrix $P_{k}$ minimizes the $\ell_{2}$-norm reconstruction error between the projected data $P_{k} P_{k}^{T} X$ and $X, X - P_{k} P_{k}^{T} X^{2}$ among all projections $P_{k} P_{k}^{T}$ to $k$ dimensional spaces. In Fig. 6, we show the explained variance as a function of the number of components. We chose $k = 30$, which resulted in $P_{k} Y$ explaining over 80% of the variance of $X$.

### Frequency bands as features of interest

Brain activity oscillations at different frequency bands have been linked to specific functions, such as attention and control mechanisms. The alpha-band (8–13 Hz) has been implicated in the inhibition of cortical regions and it has been shown to be sensitive to the attentional load [30–33]. The beta-band (13–30 Hz) is linked to top-down attention control and maintenance of function [34–36]. The gamma-band (> 30 Hz) is involved in a wide variety of conscious cognitive functions including sensory processing, attention, and executive control functions [35, 37, 38]. To extract the signals in these frequency bands we convolved $Y\left( t \right)$, the MEG sensor measurements in the training samples *T*, with complex Morlet wavelet $\psi \left( {t, f_{c} } \right)$.

i. $$ {\rm Z} = \left| {Y\left( t \right)*\psi \left( {\bar{t}, f_{c} } \right)} \right|   \in {\mathbb{R}}^{30 \times 4 \times T} ,  \quad t \in T,  f_{c} \in {\mathbb{R}} $$
  where $\psi \left( {t, f_{c} } \right)$ is defined as:
ii. $$ \psi \left( {\bar{t}, f_{c} } \right) = \frac{1}{{\sqrt {2\pi \sigma^{2} } }}e^{{ - \frac{{t^{2} }}{{2\sigma^{2} }}}} e^{{ - 2i\pi f_{c} t}} . $$

In the above formula, t is time, f_c_ is the center frequency, and σ is the bandwidth of the wavelet kernel. The four center frequencies are in the middle of the four frequency bands (8–13 Hz, 13–30 Hz, 30–60 Hz, and 60–90 Hz) of interest. Finally, *Z* is collapsed into a two-dimensional matrix *Z*$\in {\mathbb{R}}^{120 \times T}$.

### Partial least squares regression (PLS)

To identify the components that best separate the attentional states we applied partial least squares regression (PLS) to the wavelet filtered data (*Z*). PLS regression [39] is a method similar to PCA. PLS regression operates on labeled data ($y$), which, in the case of sb-NFB, is the targeted cognitive state for NFB training. The PLS algorithm is trained on labeled data from the training period of the previous block of trials. PLS algorithm can be represented as 
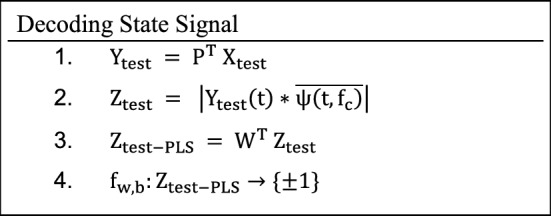


The resulting matrix *W* is used to project *Z* to a low 10-dimensional space ${\rm Z}_{\text{PLS}}$, which will then be used to train the support vector machine (SVM):

iii. $$ Z_{\text{PLS}} = W^{T} Z \in   {\mathbb{R}}^{10 \times T} $$

### The state signals

Linear support vector machine (SVM) can be described as a separating hyperplane with binary solutions on both sides (i.e., solutions equal to + 1 or − 1) whose main objective is to find a hyperplane which would minimize margin error. Linear SVMs have been used in fMRI neurofeedback studies [40]. Generally, linear SVMs are implemented to decode a behavioral state from the activity in a collection of voxels. Using this method, we decoded the targeted brain state from the collection of low dimensional PLS components. The linear SVM algorithm is trained using the PLS components during the “training” window. We used LibSVM [40] with a linear kernel and cost equal to one ($C = 1$) to compute the hyperplane $f_{w,  b} :Z_{\text{PLS}} \to \left\{ { \pm 1} \right\} | Z_{\text{PLS}} \in   {\mathbb{R}}^{10}$ parametrized by $w, b$ that best separated the attention to the left or right visual hemifields fields. The hyperplane $f_{w,  b}$ is learned during the “training” window and then evaluated in the development window to evaluate performance. Finally, Platt calibration, which fits a logistic regression model to the SVM scores, was used to transform the outputs of the SVM model into a probabilistic quantity, which we will call the state signal:$\rho (f_{w,  b} = 1 | Z_{\text{PLS}} ) = \frac{1}{{1 + \exp \left( {Af_{w,  b} \left( {Z_{\text{PLS}} } \right) + B} \right)}}$,where the parameters *A* and *B* are optimized using gradient descent to minimize the cross-entropy error [41].

### The state decoding component

To obtain the state signal, during the testing phase, through the following steps:
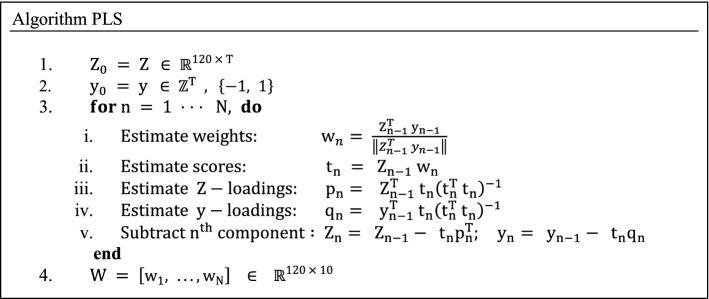


Where the data-dependent $P, W \;{\text{and}}\; f_{w,  b}$ were learned during the training phase and the data-independent wavelet kernels $\psi \left( {t,f_{c} } \right)$ remain the same as above.

The signal transformation algorithm trained in the training component is run across the time course of the trial resulting in a time course which encodes the brain state, referred to as the state signal $\left( \rho \right)$. The state signal is normalized to a $z$-score $z_{t} = {{\left( {\rho - \mu_{\rho } } \right)} \mathord{\left/ {\vphantom {{\left( {\rho - \mu_{\rho } } \right)} {\sigma_{\rho } }}} \right. \kern-0pt} {\sigma_{\rho } }}$, where $\mu_{\rho }$ is the mean of the corresponding state signal and $\sigma_{\rho }$ is its standard deviation. We used $z_{\rho } > 2$ to indicate when the subject is in the targeted brain state.

For the feedback cue in switching spatial attention (SAST), we used a red thermometer, described “Experimental protocol” and “The feedback cue” sections. The first block of trials was used to compute the initial set of switch times, and thus during the feedback thermometer was not presented. In all the following blocks of trials, the distribution of switch times from the previous block was used to compute the switch time in the current block, see “Experimental protocol” and “The feedback cue” sections. The feedback bar was not updated for catch trials.

## Data Availability

The datasets used and/or analyzed during the current study are available from the corresponding author on reasonable request.

## Appendix

The stimulus of the NFB Task consisted of two random-dot kinematograms (RDK) composed of 50% red and 50% green dots (density 2 dots/deg^2^; dot diameter 0.13°; speed 3°/s, luminance 50 cd/m^2^, background luminance 25 cd/m^2^) presented in two virtual circular apertures (6° in diameter) shown simultaneously in the LVF and RVF at equal eccentricity (8°) from a cross-hair central fixation mark (0.5° in width and height, luminance 85 cd/m^2^) displayed at the center of the screen. In both apertures, 90% of the dots, signal dots, moved in a consistent diagonal direction while the reminder dots (10%), noise dots, moved in random directions within the apertures. The red and green signal dots moved orthogonally to each other. In this study, subjects were asked to attend to the motion of the green dots.

The white fixation mark was presented constantly throughout a trial and participants were instructed to maintain fixation on it throughout the duration of the trial. After 500–1000 ms, an arrow (0.5° in width and height; luminance 85 cd/m^2^) was superimposed on the fixation cross, pointing either to the left or right hemifield. The direction of the arrow indicated the aperture to be attended to throughout the “attend” window. Concomitant with the arrow presentation, a red or green disc (1° in diameter) was shown superimposed on the RDK pattern in the non-attended aperture. The disc appeared at 10% opacity to reduce saliency, and thus not capture attention. The disc’s location within the aperture and its color (red or green) changed at random times (ranging between 300 and 600 ms) throughout the attend and switch windows of the stimulus display.

In the attended aperture, participants were instructed to pay attention to the direction of motion of the green dots. As soon as they perceived a change in direction of the attended dots, they had to switch attention to the aperture in the opposite visual field (“switch” window). In 20% of the trials (catch trials), there was no change of direction of the attended dots. In these trials, participants were instructed to not switch their attention and wait until the dots disappeared and the trial ended (1500–3000 ms). The catch trials served to control whether participants switched attention even when no cue to switch was present. In the trials that required a switch of attention to the non-attended aperture, participants were instructed to as soon as possible to the color of the disc after switching (e.g., the responses were entered with the right hand by pressing a designated button on the MEG compatible button box; “press 1 if the disc is red and 2 if the disc is green”; “response” window).

## Abbreviations

LVF: Left visual field
MVPA: Multivoxel pattern analysis
PCA: Principal component analysis
PLS: Partial least squares regression
RDK: Random-dot kinematogram
RVF: Right visual field
rt-MEG: Real-time magnetoencephalography
rt-EEG: Real-time electroencephalography
rt-fMRI: Real-time functional magnetic resonance imaging
SAST: Switching spatial attention
sb-NFB: State-based neurofeedback
SMR: Sensorimotor rhythm
SSP: Signal space projection
SVD: Singular value decomposition
SVM: Support vector machine
